# Ultrasound Grade of Liver Steatosis Is Independently Associated with the Risk of Metabolic Syndrome

**DOI:** 10.1155/2018/8490242

**Published:** 2018-08-23

**Authors:** Sanda Mustapic, Sead Ziga, Vladimir Matic, Tomislav Bokun, Bozo Radic, Marko Lucijanic, Srecko Marusic, Zarko Babic, Ivica Grgurevic

**Affiliations:** ^1^Department of Gastroenterology, Hepatology and Clinical Nutrition, University Hospital Dubrava, University of Zagreb School of Medicine, Zagreb, Croatia; ^2^Department of Emergency Medicine, University Hospital Dubrava, University of Zagreb School of Medicine, Zagreb, Croatia; ^3^Faculty of Pharmacy and Biochemistry, University of Zagreb, Croatia; ^4^Department of Hematology, University Hospital Dubrava, Zagreb, Croatia; ^5^Department of Endocrinology and Clinical Pharmacology, University Hospital Dubrava, University of Zagreb School of Medicine, Zagreb, Croatia

## Abstract

The aim of the study was to explore (a) prevalence and grade of nonalcoholic fatty liver (NAFL) among outpatients referred for abdominal ultrasound (US) examination and (b) relationship between the presence and severity of liver steatosis and metabolic syndrome (MS). This was a retrospective analysis of patients without history of liver disease examined by abdominal US in the University hospital setting. US was used to detect and semiquantitatively grade (0-3) liver steatosis. Data on patients' age, gender, body mass index (BMI), impaired glucose metabolism (IGM), atherogenic dyslipidaemia (AD), raised blood pressure (RBP), transaminases, and platelet counts were obtained from medical records. MS was defined as having at least 3 of the following components: obesity, IGM, AD, and RBP. Of the 631 patients (median age 60 years, median BMI 27.4 kg/m2, and 57.4% females) 71.5% were overweight and 48.5% had NAFL. In the subgroup of 159 patients with available data on the components of MS, patients with higher US grade of steatosis had significantly higher BMI and increased prevalence of obesity, IGM, AD, RBP, and accordingly more frequently had MS, whereas they did not differ in terms of age and gender. NAFL was independently associated with the risk of having MS in a multivariate model adjusted for age, gender, BMI, and IGM. The grade of liver steatosis did not correlate with the presence of liver fibrosis. We demonstrated worrisome prevalence of obesity and NAFL in the outpatient population from our geographic region. NAFL is independently associated with the risk of having MS implying worse prognosis.

## 1. Introduction

Nonalcoholic fatty liver disease (NAFLD) is defined as the fatty infiltration of the hepatocytes exceeding 5% of the liver weight in the absence of other causes such as excessive alcohol intake or hepatitis C [1, 2]. NAFLD encompasses a spectrum of diseases from simple hepatic steatosis (nonalcoholic fatty liver, NAFL) to steatosis with necroinflammatory changes and progressive fibrosis (nonalcoholic steatohepatitis, NASH) [3, 4]. Steatosis is generally a benign condition, whereas NASH can be associated with fibrosis, cirrhosis, and liver failure and carries a higher risk of cardiovascular disease and mortality [2, 4, 5]. NAFLD is often associated with insulin resistance, visceral obesity, excessive body mass index (BMI), type 2 diabetes mellitus (T2DM), hyperlipidemia, arterial hypertension, cardiometabolic alterations, polycystic ovarian syndrome, hypothyroidism, hypogonadism, and sleep apnea abnormalities encompassed by the common term “metabolic syndrome” (MS) [2, 6, 7]. Due to these associations, NAFLD has been until recently considered as the hepatic manifestation of MS [8, 9]. Both MS and NAFLD involve interactions of the adipokines, cytokines, inflammatory factors, and insulin resistance [9]. NAFLD is considered epidemic and serious public health issue with significant impact on the healthcare expenditures [10]. The prevalence of NAFLD is 2-44% in the general European population (including obese children) and 42.7-69.5% in people with T2DM, with an increase in prevalence with age [11]. Estimates of the worldwide prevalence of NAFLD range from 6.3 to 33% with a median of 20% in the general population [6]. Patients diagnosed with NAFLD have a higher mortality rate when compared to the general population of the same sex and age because of the increased prevalence of cardiovascular disease and increased liver-related mortality rate [12]. A German study showed that subjects with NAFLD as detected by ultrasound (US) and increased serum alanine aminotransferase (ALT) levels had 26% higher overall healthcare costs at 5-year follow-up [13]. Although liver biopsy is the gold standard for diagnosing NAFLD [14], it is not the method of choice to be used in studies that involve general population due to its invasiveness and costs. US, as an alternative tool, is noninvasive, relatively inexpensive, and widely available and increasingly accepted as a method for the initial screening of patients suspected to have NAFLD. According to a meta-analysis, US has pooled sensitivity of 84.8% and specificity of 93.6%, with area under the receiver operating characteristic (ROC) curve of 0.93, to detect moderate-to-severe steatosis (fatty infiltration of >20-30% hepatocytes) [15, 16]. In terms of quantification of liver steatosis, US has recently been challenged by controlled attenuation parameter (CAP), another noninvasive method based on transient elastography (TE) [17, 18]. It has been debated whether the presence and grade of steatosis are to be considered “just” an additional component of the MS or if they have a potential role in the development of MS with prognostic implications [9, 19].

Therefore, the main goals of our study were (a) to assess the prevalence and grade of NAFLD among the cohort of outpatients referred for an US examination and (b) to explore the relationship between the presence and severity of liver steatosis and MS.

## 2. Patients and Methods

### 2.1. Study Population

In this study, retrospective analysis was performed over the cohort of consecutive outpatients examined by 3 experienced ultrasonographers in the US Unit of the University Hospital Department of Gastroenterology during a 5-month period.

The study was approved by the Institutional Review Board and was performed in line with the ethical guidelines of the 1975 Helsinki declaration. Upon this approval the following data were retrieved from the patients' medical records: age, sex, BMI, medical history including the history of liver disease, malignancy, diabetes and other forms of impaired glucose metabolism, dyslipidaemia, hypertension, and patients' medications were reviewed looking for the known causes of liver steatosis. All patients gave their verbal consent before US examination. According to national policy only verbal consent is required for US examination provided that no invasive procedures are to be performed.

The exclusion criteria were presence of previously defined hepatobiliary disease other than NAFLD, malignancies, ascites, the use of medications (current or within the last 12 months) known to induce hepatic steatosis (estrogens, corticosteroids, amiodarone, valproate), inflammatory bowel disease, and human immunodeficiency virus (HIV) infection. The following biochemical parameters were documented as well: bilirubin, aspartate aminotransferase (AST), ALT, gamma-glutamyl transferase (GGT), alkaline phosphatase (ALP), and platelet count within 3 months form the US examination. These parameters were used to calculate Fibrosis-4 (FIB4) score as a noninvasive indicator of liver fibrosis stage, since association between severity of liver steatosis and fibrosis has been investigated, and liver fibrosis has been confirmed the most important histological factor in terms of overall mortality and liver-related outcomes [20, 21]. We used dichotomized cut-off values optimized to rule-in (FIB4 ≥2.67; PPV 80%) and rule-out (FIB4≤1.3; NPV 90%) advanced (F≥3) fibrosis [22].

**Figure 1:**
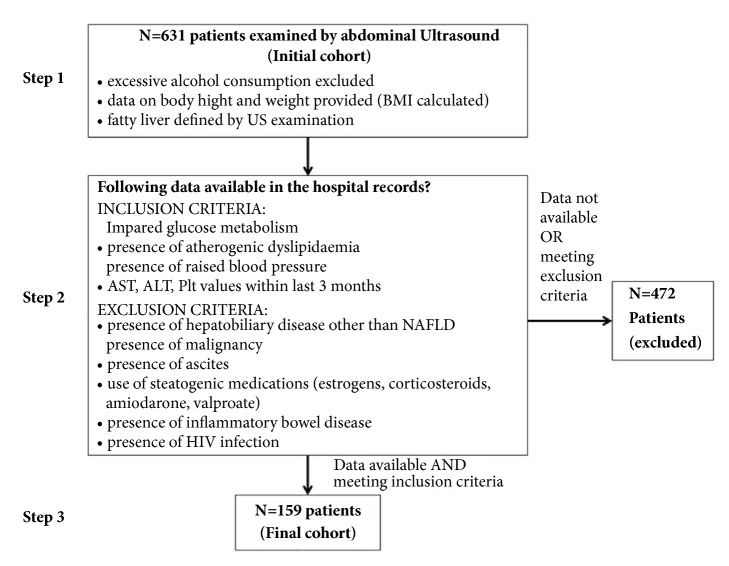
Study protocol. N: number of patients; BMI: body mass index; US: ultrasound; AST: aspartate transaminase; ALT: alanine aminotransferase; Plt: platelet count; NAFLD: nonalcoholic fatty liver disease; HIV: human immunodeficiency virus.

Given the fact that analysis of previous US results demonstrated increasing trend of fatty liver reported in our Unit, we adopted the policy to collect information on patients' body weight and height (parameters needed to calculate BMI) as routine part of the US examination protocol for the purpose of the internal quality control. In addition to this, patients that were found to have fatty liver were routinely asked to quantify their alcohol intake in order to exclude confounding factors in the pathogenesis of hepatic steatosis. We consider excessive alcohol consumption as drinking an intake >40 g/day for men and >20 g/day for women. Therefore, data on patients' age, sex, BMI, alcohol intake, and US findings were available for all patients encompassed by this analysis (N=631, Initial cohort). Since many patients have just undergone abdominal US in our hospital without any further consultancies or blood tests, the number of patients with all parameters available as defined by the study protocol was considerably lower (N=159, Final cohort) than the total number of patients encompassed by initial examination. Study protocol is depicted at Figure 1.

For the purpose of this study we used modified definition of MS as the presence of at least 3 out of 4 of the following components: central obesity, atherogenic dyslipidaemia (AD), raised blood pressure (RBP), and impaired glucose metabolism (IGM). All these have been widely accepted as core components by various definitions of MS in use (World Health Organization (WHO) [23], the European Group for the Study of Insulin Resistance (EGIR) [24], the National Cholesterol Education Program-Third Adult Treatment Panel (NCEP ATP III) [25], and International Diabetes Federation (IDF) [26]). The common components in each of these definitions that are used in this study are defined as follows: (1) central obesity: waist circumference with ethnicity-specific values OR BMI>30 kg/m^2^, in which cases central obesity can be assumed and waist circumference does not need to be measured); (2) AD: raised triglycerides (TG): > 150 mg/dL (1.7 mmol/L) OR reduced high-density lipoprotein (HDL) cholesterol: < 40 mg/dL (1.03 mmol/L) in males, < 50 mg/dL (1.29 mmol/L) in females, OR specific treatment for these lipid abnormalities; (3) RBP: systolic BP > 130 or diastolic BP >85 mm Hg, or treatment of previously diagnosed hypertension; (4) IGM: raised fasting plasma glucose (FPG): >100 mg/dL (5.6 mmol/L), or previously diagnosed T2DM. For the prospective study, sticking to the one of these definitions would certainly be more appropriate. However, since this was retrospective analysis form the available medical records, and some data would have been missing to fit into some of these definitions, we decided to adopt a more a general approach in order to ascertain enough statistical power for the study.

### 2.2. US Assessment

Abdominal US was performed in patients after overnight fasting, in supine position or in the left decubital position to achieve the best possible visualization of the liver. The US probe was lubricated with gel to avoid artifacts from air and dry skin surfaces. Each US exam was performed by one of three physicians with extensive US experience using an Aixplorer® Ultrasound system, equipped with convex transducer XC6-1 with a 1-6 MHz bandwidth (Supersonic Imagine, Aix-en-Provence, France). Fatty liver on US displayed in the grey scale looks brighter relative to the kidney cortex. With the increased accumulation of liver fat, US waves become more attenuated resulting in a decreased visualization of the deeper parts of the liver (diaphragm and hepatic veins). Diagnosis of fatty liver was based on the increased echogenicity of the liver parenchyma as compared to the right kidney's cortex. Visibility and sharpness of the diaphragm and hepatic veins' interface were analyzed as well. Based on these 3 parameters steatosis was further classified into 3 grades: Grade 0, no steatosis (liver and renal cortex of the same echogenicity); Grade 1, mild steatosis: slightly brighter liver as compared to the renal cortex, clear visualization of diaphragm, and interface of hepatic veins with sharp contours; Grade 2, moderate steatosis: brighter liver with attenuated US beam at deeper parts of the liver, diaphragm, and hepatic veins still visible but with blunted contours; Grade 3, severe steatosis: very bright liver, severe US beam attenuation, diaphragm, or hepatic veins not visible. This classification was adopted and already tested by other investigators [27].

### 2.3. Statistical Analysis

Normality of distribution of numerical variables was tested using the Kolmogorov-Smirnov test. All numerical variables were nonnormally distributed and were summarized as median and interquartile range (IQR). The Mann–Whitney* U* test/the Kruskal-Wallis analysis of variance (ANOVA) test were used to compare numerical variables between groups where appropriate. Categorical variables were summarized as number and percentage. Jonckhere Terpstra test for trend and the (Chi squared) Χ^2^ test for trend were used to assess trends of increase of tested variables among rising grades of steatosis.

The Χ^2^ test was used to compare categorical variables between groups. The logistic regression was used to investigate associations of categorical variables with other variables while adjusting for potential confounders. P values <0.05 were considered to be statistically significant. Analyses were done using MedCalc Statistical Software version 17.6 (MedCalc Software bvba, Ostend, Belgium).

## 3. Results

### 3.1. Demographic Characteristics of Study Participants

A total of 631 patients were encompassed by the primary analysis. There were 269 (42.6%) males and 362 (57.4%) females; median age was 60 years, IQR (49 – 69.75) years, and median BMI was 27.4, IQR (24.6 – 30.65). Excessive body weight (BMI ≥25 kg/m^2^) was recorded in 451/631 (71.5%) patients (41.4% with a BMI 25-29.9 kg/m^2^ (overweight) and 30.1% BMI >30 kg/m^2^ (obese)). Fatty liver was found in 306/631 (48.5%) patients (US grade 1 in 29.3%, grade 2 in 16.3%, and grade 3 in 2.9% of all patients, with no significant difference between males and females). Demographic characteristics of the patients are presented in Table 1. Patients with fatty liver (N=306) were significantly older (median 61 versus 59 years, p=0.028) and had significantly higher BMI (median 29.7 versus 25.5, p<0.001), but had no difference in terms of gender (45.8% males versus 39.7% females, p=0.124). Prevalence of obesity was significantly higher among patients with, as compared to those without fatty liver (23.1% versus 6.9%, respectively, p<0.001).

### 3.2. Relationship between Fatty Liver and Metabolic Syndrome

In the second step, we explored the prevalence and the relationship between the components of MS and the presence and US grade of fatty liver.

For the initial cohort of patients (N=631), only the analysis on the relationship between the US grade of liver steatosis and BMI was possible revealing significant increase in BMI over rising grades of liver steatosis (median BMI values of 25.5, 27.8, 31.5, and 36.5 kg/m^2^ for grades 0 to 3, respectively, P<0.001). In the post hoc analyses, BMI was significantly different between each grade of steatosis (P<0.05 for all comparisons).

**Figure 2:**
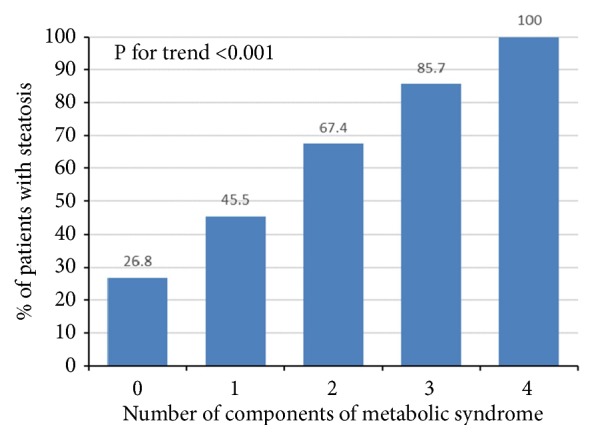
The prevalence of nonalcoholic fatty liver disease (NAFLD) as detected by ultrasound in relationship to the number of components of metabolic syndrome (final cohort, N=159 patients). There is a statistically significant trend of increase in proportion of NAFLD among patients with rising number of metabolic syndrome components, the Χ^2^ test for trend, P<0.001.

Reliable data on the existence of AD, IGM, and RBP were available for 182 patients, of which 23 met exclusion criteria, and the remaining 159 that formed the final cohort had no conditions known to be capable of inducing fatty liver transformation (see exclusion criteria in Patients and Methods section and Figure 1). Thirty-seven percent of patients in the final cohort were males and 62.3% females, with median age of 59 years, IQR (48.5 – 67.5), and median BMI of 27.3 kg/m^2^, IQR (24.4 - 31.5) (69.2% overweight and 34% obese). These results were similar to the initial cohort of 631 patients. AD was present in 65/159 (40.9%), IGM in 34/159 (21.4%), and RBP in 80/159 (50.3%) of patients. In the final cohort, 88 of 159 (53.4%) had fatty liver: 52/88 (59.1%) grade 1, 28/88 (31.8%) grade 2, and 8/88 (9.1%) grade 3. Since only 8 patients had US grade 3 of liver steatosis, for the purpose of further analysis these were merged with patients having US grade 2 steatosis in a single group named “moderate-to-severe steatosis”. Fatty liver was detected in 72/110 (65.5%) overweight patients, 26/34 (76.5%) patients with IGM, 47/65 (72.3%) patients with AD, and 56/80 (70%) with RBP. There were no significant differences in age and gender between patients with different US grades of liver steatosis, nor did they significantly differ in liver function tests (Table 2). However, patients with higher US grade of steatosis had significantly higher BMI, increased prevalence of obesity, IGM, AD, and RBP and accordingly significantly more frequently met the modified definition of MS as used in this study (P<0.05 for all analyses) (Table 2). The probability of having NAFLD increased proportionally to the number of components of the MS present in the individual patient (P<0.001) (Figure 2).

US grade of liver steatosis was significantly independently associated with the presence of MS in a series of multivariate models (multiple logistic regression Model 1: nonadjusted model; Model 2: adjusted for age and sex; Model 3: additionally adjusted for BMI; Model 4: additionally adjusted for IGM) as shown in Table 3. Patients with mild liver steatosis had odds ratio (OR) of 5.13 (P=0.07), while patients with moderate-to-severe liver steatosis had OR of 14.68 (P=0.007) for having MS after accounting for aforementioned confounding variables.

**Figure 3:**
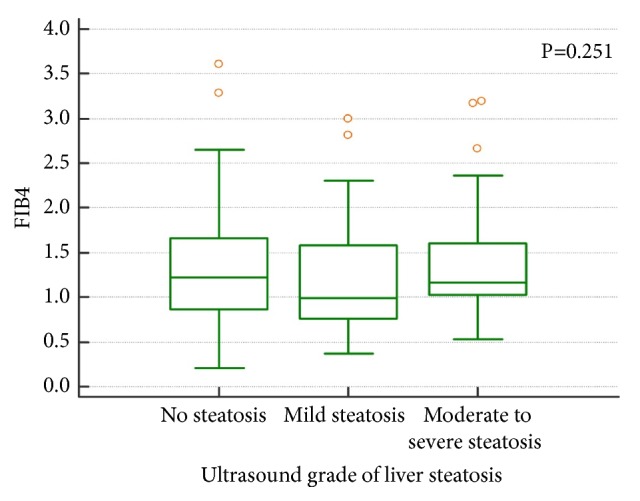
Fibrosis-4 (FIB4) score did not significantly differ between different grades of liver steatosis, the Kruskal-Wallis ANOVA test, P=0.251.

Median FIB4 for the final cohort (N=159) was 1.2, IQR (0.8 - 1.6). Only 6/159 (3.8%) patients had FIB4 ≥2.67 indicative of advanced liver fibrosis, 98/159 (61.6%) had FIB4≤1.3, while 55/159 (34.6%) remained between the two values and it was thus not possible to reliably classify them into risk categories for fibrosis. There was no significant difference in individual FIB4 category between patients with and without fatty liver (52/88 (59.1%) patients with fatty liver and 40/71(56.3%) without fatty liver had FIB4≤1.3). Accordingly, there was no significant difference in FIB4 value between different US grades of liver steatosis (p=0.251) (Figure 3).

## 4. Discussion

In this study, we demonstrated worrisome prevalence of overweight, obesity, and NAFLD in outpatient population from our geographic region. These results are also indicative of the association between higher US grades of liver steatosis and increased risk of having MS and this association was independent from the confounding variables such as age, gender, BMI, and IGM. No significant difference in terms of noninvasively assessed liver fibrosis using FIB4 could be demonstrated between different grades of liver steatosis.

The first important finding of this study is very high prevalence of overweight (41.4%), obesity (30.1%), and NAFL (48.5%) in the analyzed population. These results reveal even worse figures as compared to those reported in the recent European survey showing the prevalence of overweight in 47.6% and obesity in 12.8% of European adults, with respective figures in Croatia being 36.7% and 21.5% [28]. To put these data in the regional context of Central European countries, the corresponding figures of the prevalence of overweight/obesity are 55.6%/9.8% for Hungary, 34.0%/10.5% for Austria, 34.6%/11.7% for Czech Republic, 34.1%/25.4% for Slovakia, and 37.5%/10.3% for Poland [28, 29]. The difference in the reported prevalence of overweight and obesity in Croatian population between the European survey and this study might probably be largely attributed to the selection bias, since our patients are not ideal representatives of the general population. In fact, all of them have been referred to US examination for some medical condition, and it has been well appreciated that overweight and obesity raise the risk for morbidity and overall mortality [30]. Conversely, it is logical to expect higher prevalence of overweight and obesity in people suffering from various health conditions as compared to their lean counterparts. Nevertheless, these figures are worrisome and call for wider action in our community to tackle this growing epidemiological problem. On the other hand, prevalence of MS in our cohort was “only” 19.5%, in contrast to the figures obtained for overweight and obesity. The prevalence of MS is obviously underestimated as anticipated from the study design and especially due to modified definition for MS used here which represents limitation.

The other important finding of this study is that NAFLD patients represent cohort under significant health risk since they have not only high BMI, but also significantly higher prevalence of other individual components of MS. In fact, worsening grades of liver steatosis as detected by US are accompanied by increased prevalence of more individual components and MS itself (Table 3).

Accordingly, patients with more components of MS had higher prevalence of NAFLD (Figure 2). Association between NAFLD and MS has been recognized before and NAFLD was even considered hepatic manifestation of MS [31]. This concept has been questioned recently as the longitudinal studies provided evidences that NAFLD preceded development of MS [19]. In the elegant population-based cross-sectional study coming from Taiwan, in which another semiquantitative US scoring system was used to grade liver steatosis, the authors demonstrated independent association of liver steatosis and the MS, after adjustment for BMI and insulin resistance as assessed by HOMA-IR [9].

Namely, patients with higher US grades of liver steatosis had increasingly higher OR for MS (3.64 and 9.4 respectively for those with mild and moderate- to-severe NAFLD, as compared to those without NAFLD), which is in line with results obtained in our study. In our cohort association of liver steatosis and MS was consistent over different grades of disease severity in a series of multivariate models adjusted for age, gender, BMI, and IGM. We acknowledge that the result for mild grade steatosis in our final model was of borderline statistical significance. However, clear trend in favor of increased odds of MS with mild disease and clear association of moderate-to-severe disease with MS in our final model are evident. Although limited by retrospective analysis of our data, our study replicates and further supports current concepts of fatty liver as an independent risk factor for MS [9, 19]. In addition, NAFL was found in patients who did not fulfill the criteria of MS and might predict the development of MS or might be a separate pathological entity characterized by a specific genetic predisposition. The latter observation is speculative but might be of use to further investigation of a specific subgroup of patients in future studies.

Concerning the clinical utility of transabdominal US, our data provide evidence that simple semiquantitative scoring of liver steatosis by US reliably predicts severity of metabolic derangements as defined by the increasing number of components of MS. If NAFLD precedes development of MS, its detection would imply the necessity for correction in the early stage by potentially simple intervention such as weight loss. In case when higher grade of steatosis was detected, more comprehensive diagnostic work-up is mandatory to assess the presence and severity of MS. The main limitation of US is the lack of sensitivity to diagnose mild steatosis, as it is capable of diagnosing fatty liver only when at least 20% of hepatocytes have been fatty transformed [16]. Along these lines it should be noted that levels of commonly used biochemical tests such as aminotransferase were not significantly different between patients with different grades of liver steatosis and therefore are obviously not useful for predictive purposes which has already been demonstrated by other authors as well [32, 33].

The final observation from our study is that higher grade of liver steatosis does not impose significant risk for liver fibrosis. This finding should be interpreted with caution due to design of our study and since we used single noninvasive parameter with 1/3 of patients being classified within the grey zone between the values that reliably rule-in (≥2,67) and rule-out (≤1.3) advanced (F≥3) fibrosis. According to current recommendations, two unrelated noninvasive tests (one usually based on US elastography and one biochemical) should be applied to assess the stage of liver fibrosis [34]. In case of disagreement between the two, biopsy is advisable if the result would influence further management of the patient. This concept is important since noninvasive tests are not without limitations. For example, it has been recently demonstrated that higher CAP categories (i.e., more steatotic liver, CAP>300 dB/m) influence the results of liver stiffness measurement (LSM) leading to overestimation of fibrosis especially in the lower range of the fibrosis spectrum [35]. This might have been the reason why significant fibrosis (defined as LSM≥7 kPa) and even cirrhosis (LSM≥10.3 kPa) were observed with a prevalence of 16.7-18.8% and 13.8%, respectively, as reported by the authors who used transient elastography and CAP to assess liver steatosis and fibrosis [36, 37]. It should be also noticed that LSM cut-off values for significant fibrosis and cirrhosis as measured by TE are not the same for different etiologies of liver diseases [38]. By using FIB4, a representative of biochemical tests, the prevalence of advanced fibrosis (F≥3) in our cohort was “only” 3.8%. It is hard to compare these results since we were looking for advanced fibrosis (F≥3), and the other two studies were focused on significant fibrosis and cirrhosis and the inclusion criteria were different.

Furthermore, discrepancies between the reported prevalence of advanced fibrosis/cirrhosis in patients with T2DM (5-7% in UK and 13.8% in Romania) might be at least to some extent attributed to the different methods used to assess liver fibrosis, i.e., biochemical NAFLD fibrosis score (NFS) in the former and TE with CAP in the latter study [39–41]. Our results are in line with the currently prevailing concept that the amount of liver fat is not predictive for the risk of having liver fibrosis [20]. However, this concept has been based mostly on cross-sectional studies, whereas data from the longitudinal studies reveal that gaining the weight and accumulating more liver fat on follow-up biopsy are connected to the higher risk of fibrosis progression [20]. On the other hand, in the Rotterdam study that included 3,041 participants from the general population, steatosis as detected by US was strongly associated with the presence of clinically relevant fibrosis (defined as LSM≥8 kPa by TE [42], with the prevalence of 5.6%). Since no histology data were provided, these results might suffer from the same limitation as previously mentioned studies due to the possible overestimation of fibrosis in patients with more severe steatosis.

Apart from liver-related risks, higher grades of liver steatosis are related to the increased risk of cardiovascular morbidity [43, 44].

The main limitation of our study is its retrospective approach. For this reason certain data that were needed to meet one of the established definitions of MS were not available from the medical records precluding formation of the uniform cohort with significant power to perform statistical analysis. Thus, for the purpose of this study, MS was considered in patients who had at least 3 (any 3) out of 4 core components contained in any established definition of MS. Since dyslipidaemia was defined by either elevated TG or low HDL values or specific medication used for these conditions (similar to WHO and EGIR definition), it might have underestimated the real prevalence of MS, as cases with both lipid components were not recognized by this definition. Along this line, overlapping between high TG and low HDL might have been presumed especially in obese patients already commenced to lipid-lowering therapy, even though this overlapping was not evident from the biochemical data available at the study inception. Further limitation refers to the liver biopsy which was not available, and our results rely on previous studies that demonstrated good correlation between the US and histological diagnosis and grading of liver steatosis. The same limitation stands for the assessment of liver fibrosis, which was performed by calculating only FIB4 score in our study.

In conclusion, results of this study demonstrate high prevalence of overweight, obesity, and NAFLD in the outpatient population in our geographic region.

Patients with higher US grades of liver steatosis are under increased risk of MS independently of age, gender, BMI, and IGM, but not of liver fibrosis. Simple semiquantitative US scoring of liver steatosis might help in earlier recognition of MS and enable timely interventions aimed to reduce cardiovascular risk, therefore improving prognosis of these patients.

## Figures and Tables

**Table 1:** Demographic and US findings of the entire examined cohort (N=631 patients).

Age (years) (median, IQR)	60 (49-69.75)
Sex (M/F) (N, %)	269 (42.6%) / 362 (57.4%)
Body weight (kg) (median, IQR)	78 (68 - 89)
Height (cm) (median, IQR)	168 (162 - 175)
BMI (kg/m^2^) (median, IQR)	27.4 (24.6 - 30.7)
BMI >25 kg/m^2^ (N, %)	451 (71.5%)
*BMI 25-29.9 kg/m* ^*2*^	261 (41.4%)
*BMI >30 kg/m* ^*2*^	190 (30.1%)
Liver steatosis (any US grade) (N, %)	306 (48.5%)
*US grade 1 steatosis *	185 (29.3%)
*US grade 2 steatosis *	103 (16.3%)
*US grade 3 steatosis*	18 (2.9%)

IQR: interquartile range; US: ultrasound; BMI: body mass index.

**Table 2:** Relationship between ultrasound grade of liver steatosis and clinical features of patients from the final cohort (N=159).

	**Normal liver**	**Mild steatosis**	**Moderate to severe steatosis**	***P*-value for trend**
Number	71	52	36	-
Age, years (median, IQR)	60 (47 - 69.5)	56.5 (48 - 67.5)	59.5 (53.25 - 65.25)	0.862
Sex (Male) (N, %)	23 (32.4%)	22 (42.3%)	15 (41.7%)	0.281
BMI, kg/m^2^ (median, IQR)	25.4 (22.9 - 27.8)	28.5 (25.2 - 32)	32.6 (28.02 - 35.85)	<0.001*∗*
Obesity (N, %)	11 (15.5%)	21 (40.4%)	22 (61.1%)	<0.001*∗*
IGM (N, %)	8 (11.3%)	14 (26.9%)	12 (33.3%)	0.005*∗*
Atherogenic dyslipidaemia (N, %)	18 (25.4%)	25 (48.1%)	22 (61.1%)	<0.001*∗*
Raised blood pressure (N, %)	24 (33.8%)	33 (63.5%)	23 (63.9%)	0.001*∗*
Presence of MS (N, %)	3 (4.2%)	12 (23.1%)	16 (44.4%)	<0.001*∗*
Number of components of MS (median, IQR)	1 (0 - 1)	2 (1 - 2)	2 (1 - 3)	<0.001*∗*
AST (IU/L) (median, IQR)	22 (17.5 - 24)	21 (15.8 - 26.5)	24 (20 - 30.25)	0.096
ALT (IU/L) (median, IQR)	19 (13 - 24.5)	20.5 (16 - 26.8)	20.5 (17 - 29.5)	0.051
Bilirubin (mmol/L)	12 (10 - 14)	13.4 (10.3 -	13.1 (10.05 -	0.115
(median, IQR)		15.2)	15.375)	
GGT (IU/L) (median, IQR)	22 (15 - 35)	24.5 (17.8 - 38.5)	25.5 (18.75 - 33.5)	0.083
ALP (IU/L) (median, IQR)	68 (57.5 - 78)	71.5 (60 - 84)	71.5 (56 - 86.25)	0.334
Plt (x10^9^/L) (median, IQR)	234 (198.5 - 274)	244 (200.3 - 318)	251 (218.25 - 301)	0.169
FIB4 (IU/L) (median, IQR)	1.2 (0.9 - 1.7)	1 (0.8 - 1.5)	1.2 (1.026 - 1.587)	0.739

*∗*statistically significant at P<0.05

BMI: body mass index; dyslipidemia: triglycerides>upper limit of normal or high-density lipoprotein (HDL)<lower limit of normal; MS: metabolic syndrome; obesity: BMI>30 kg/m2; IQR: interquartile range; IGM: impaired glucose metabolism; AST: aspartate aminotransferase; ALT: alanine aminotransferase; GGT: gamma-glutamyl transferase; ALP: alkaline phosphatase; Plt: platelet count, FIB4: fibrosis-4 index, RBP: raised blood pressure. Mild steatosis: ultrasound grade 1; moderate to severe steatosis: ultrasound grades 2 and 3.

**Table 3:** Odds ratios (ORs) for metabolic syndrome regarding degree of NAFLD.

**Mild NAFLD**		**Moderate to severe NAFLD**
Model 1	OR 6.8	OR 18.13
	95% C.I. [1.81 – 25.56]	95% C.I. [4.8 – 68.57]
	P=0.005*∗*	P<0.001*∗*

Model 2	OR 7.95	OR 20.58
	95% C.I. [2.03 – 31.13]	95% C.I. [5.2 – 81.43]
	P=0.003*∗*	*P*<0.001*∗*

Model 3	OR 5.31	OR 9.89
	95% C.I. [1.29 – 21.93]	95% C.I. [2.22 – 44]
	P=0.021*∗*	P=0.003*∗*

Model 4	OR 5.13	OR 14.68
	95% C.I. [0.89 – 19.71]	95% C.I. [2.08 – 103.67]
	P=0.070	P=0.007*∗*

*∗*statistically significant at P<0.05

## Data Availability

The raw data obtained by the authors which were used for all calculations and the results of this study were submitted as a supplementary file with the manuscript.
